# Parity-time symmetry in coherent asymmetric double quantum wells

**DOI:** 10.1038/s41598-019-39085-6

**Published:** 2019-02-22

**Authors:** Si-Cong Tian, Ren-Gang Wan, Li-Jie Wang, Shi-Li Shu, Huan-Yu Lu, Xin Zhang, Cun-Zhu Tong, Min Xiao, Li-Jun Wang

**Affiliations:** 1 State Key Laboratory of Luminescence and Applications, Changchun Institute of Optics, Fine Mechanics and Physics, Chinese Academy of Sciences, Changchun, 130033 China; 2 School of Physics and Information Technology, Shaanxi Normal University, Xi’an, 710062 China; 3 Department of Physics, University of Arkansas, Fayetteville, Arkansas 72701 USA; 4 University of Chinese Academy of Sciences, Beijing, 100049 China

## Abstract

A coherently prepared asymmetric double semiconductor quantum well (QW) is proposed to realize parity-time (*PT*) symmetry. By appropriately tuning the laser fields and the pertinent QW parameters, *PT*-symmetric optical potentials are obtained by three different methods. Such a coherent QW system is reconfigurable and controllable, and it can generate new approaches of theoretically and experimentally studying *PT*-symmetric phenomena.

## Introduction

The non-Hermitian parity-time (*PT*)-symmetric Hamiltonians, which were firstly proposed by Bender and Boettcher in 1998, have attracted great attention^1^. Because of the isomorphism between quantum Schrödinger equations and paraxial-wave equations^2^, optical system becomes an ideal bed for experimentally studying *PT* symmetry. By balancing gain and loss, optical *PT* symmetry has been realized in different coupled structures, such as waveguides^3,4^, lattices^5,6^, micro-cavities^7,8^, and can be used in the fields of unidirectional propagating^9,10^, perfect absorbers^11,12^, photon lasers^13,14^, phonon lasers^15^ and sensors^16^. In addition, several interesting phenomena, such as optical solitons^17^, Bloch oscillations^18^ and topological insulators^19^ have also been investigated in optical *PT*-symmetric systems. However, most of the work, particularly in experiment, was based on solid-state materials. Once the structures are fabricated, the properties such as the threshold of the system are unable to be changed.

The susceptibility of host semiconductor quantum wells (QWs) can be controlled via electrical field^20–22^, carrier density^23^ and laser field^24^. The optical response of QWs is significantly enhanced when the frequency of light field is near resonance with the intersubband transition which has large dipole matrix element^25^. As a result, one can obtain a dramatic change in the complex dielectric constant^20–25^. Combined with electromagnetically induced transparency (EIT)^26,27^, both the refractive index and the absorptive coefficient of QWs can be effectively manipulated via atomic coherence and quantum interference.

In this work, we propose to use a coherently prepared asymmetric double semiconductor QWs to obtain *PT* symmetry. Such QW systems have been proved to have the possibility to realize quantum coherence and interference^28–30^. We demonstrate that *PT*-symmetric optical potentials, such as coupled optical waveguides, one-dimensional (1D) and two-dimensional (2D) *PT*-symmetric optical lattices can be realized by different methods. Besides, it is possible to control the *PT*-symmetric properties by tuning the laser fields and the QW parameters. The realization of *PT* symmetry in coherent QW systems by laser fields have several advantages. Firstly, compared with solid-state materials with micro-structures or nano-structures, *PT*-symmetric properties in this system can be established by different methods, and can be effectively controlled by various parameters. Secondly, *PT* symmetry has been constructed theoretically^31–34^ and experimentally^35^ in different atomic systems. Compared with these atomic systems, semiconductor QW systems have designable and flexible of energy levels, and they are easy to be integrated and stable for practical application. Thirdly, in such systems large nonlinearity can be realized assisted by EIT^36–38^, which makes it possible to observe traveling effects of lights in non-Hermitian nonlinear optical systems^39–41^.

## Models and Equations

**Figure 1:**
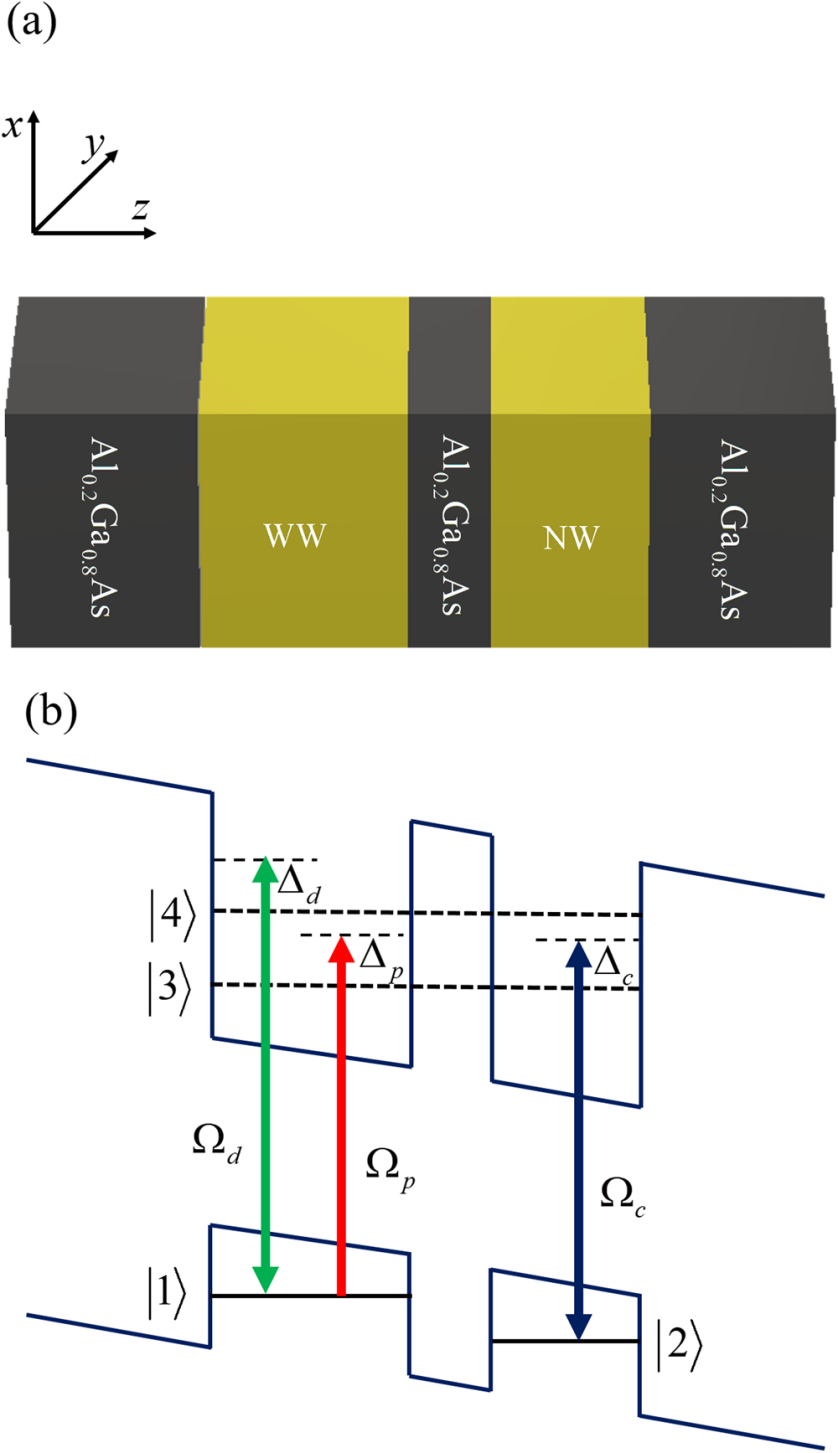
(**a**) Schematic of one pair of asymmetric double QWs with buffer layers. (**b**) Band diagram of the asymmetric double QWs, and *z* represent the wafer-growth direction.

We consider asymmetric double semiconductor QWs^42^, which consist of a 51-monolayer (145 Å)-thick wide well (WW) and 35-monolayer (100 Å)-thick narrow well (NW). Between them there is a thickness of a 9-monolayer (25-Å)-thick Al_0.2_Ga_0.8_As barrier, as shown in Fig. 1(a). There are ten pairs of QWs (each pair consists of one WW, one NW, and one thick barrier), which are isolated from each other by 200-Å-wide Al_0.2_Ga_0.8_As buffer layers. All these pairs are sandwiched between nominally undoped 3500-Å-thick Al_0.2_Ga_0.8_As layers.

Such asymmetric double semiconductor QWs can be treated as a four-level N configuration^28–30^, as shown in Fig. 1(b). Here, levels |1〉 and |2〉 are localized hole states in a valence band, while levels |3〉 and |4〉 are delocalized bonding and antibonding states in a conduction band, arising from the tunneling effect between the WW and NW via the thin barrier, respectively. The probe field *E*_p_ with frequency *ω*_*p*_ probes the transition $|1\rangle \leftrightarrow |3\rangle$, while the coupling field *E*_c_ with frequency *ω*_*c*_ and the pump field *E*_d_ with frequency *ω*_*d*_ act on transitions $|2\rangle \leftrightarrow |3\rangle$ and $|1\rangle \leftrightarrow |4\rangle$ respectively. The Rabi frequency of the probe, coupling, and pump fields are Ω_*p*_ = *μ*_13_*E*_*p*_/2h, Ω_*c*_ = *μ*_23_*E*_*c*_/2h, and Ω_*d*_ = *μ*_14_*E*_*d*_/2h, respectively, where *µ*_*ij*_ is the associated dipole transition matrix element, and the detuning of the probe, coupling, and pump fields are Δ_*p*_ = *ω*_*p*_ − *ω*_31_, Δ_*c*_ = *ω*_*c*_ − *ω*_32_, and Δ_*d*_ = *ω*_*d*_ − *ω*_42_, respectively, where *ω*_*ij*_ is the transition frequency between levels |*i*〉 and |*j*〉.

Under the condition of low QW carrier intensity, many-body effects are attributed to electron–electron interactions can be neglected^43^. In the interaction picture and under the rotating wave approximation, the Hamiltonian of the QW system can be written as (h = 1)

1 $$\begin{array}{rcl}H & = & ({{\rm{\Delta }}}_{c}-{{\rm{\Delta }}}_{p})|2\rangle \langle 2|-{{\rm{\Delta }}}_{p}|3\rangle \langle 3|-{{\rm{\Delta }}}_{d}|4\rangle \langle 4|\\  &  & +\,[{{\rm{\Omega }}}_{p}|1\rangle \langle 3|+{{\rm{\Omega }}}_{c}|2\rangle \langle 3|+{{\rm{\Omega }}}_{d}|1\rangle \langle 4|+{\rm{H}}{\rm{.c}}{\rm{.}}].\end{array}$$

Here, H.c. is the Hamiltonian complex conjugate.

The equation of motion for the density matrix of the system under the relaxation process is

2 $$\dot{\rho }=-\,i[H,\rho ]-\frac{1}{2}\{\gamma ,\rho \},$$

where *γ* is the dissipation matrix. Substituting Eq. (1) into Eq. (2), the density matrix for each element can be obtained:

3a $${\dot{\rho }}_{22}=i{{\rm{\Omega }}}_{c}({\rho }_{32}-{\rho }_{23})+{{\rm{\Gamma }}}_{42}{\rho }_{44}+{{\rm{\Gamma }}}_{32}{\rho }_{33}-{{\rm{\Gamma }}}_{2}{\rho }_{22},$$

3b $${\dot{\rho }}_{33}=i{{\rm{\Omega }}}_{c}({\rho }_{23}-{\rho }_{32})+i{{\rm{\Omega }}}_{p}({\rho }_{13}-{\rho }_{31})-{{\rm{\Gamma }}}_{3}{\rho }_{33}+{{\rm{\Gamma }}}_{43}{\rho }_{44},$$

3c $${\dot{\rho }}_{44}=i{{\rm{\Omega }}}_{d}({\rho }_{14}-{\rho }_{41})-{{\rm{\Gamma }}}_{4}{\rho }_{44},$$

3d $${\dot{\rho }}_{12}=-\,i{{\rm{\Omega }}}_{c}{\rho }_{13}+i{{\rm{\Omega }}}_{p}{\rho }_{32}+i{{\rm{\Omega }}}_{d}{\rho }_{42}-{\tilde{\gamma }}_{12}{\rho }_{12},$$

3e $${\dot{\rho }}_{13}=-\,i{{\rm{\Omega }}}_{c}{\rho }_{12}+i{{\rm{\Omega }}}_{d}{\rho }_{43}+i{{\rm{\Omega }}}_{p}({\rho }_{33}-{\rho }_{11})-{\tilde{\gamma }}_{13}{\rho }_{13},$$

3f $${\dot{\rho }}_{14}=i{{\rm{\Omega }}}_{p}{\rho }_{34}+i{{\rm{\Omega }}}_{d}({\rho }_{44}-{\rho }_{11})-{\tilde{\gamma }}_{14}{\rho }_{14},$$

3g $${\dot{\rho }}_{23}=-\,i{{\rm{\Omega }}}_{p}{\rho }_{21}+i{{\rm{\Omega }}}_{c}({\rho }_{33}-{\rho }_{22})-{\tilde{\gamma }}_{23}{\rho }_{23},$$

3h $${\dot{\rho }}_{24}=-\,i{{\rm{\Omega }}}_{d}{\rho }_{21}+i{{\rm{\Omega }}}_{c}{\rho }_{34}-{\tilde{\gamma }}_{24}{\rho }_{24},$$

3i $${\dot{\rho }}_{34}=i{{\rm{\Omega }}}_{p}{\rho }_{14}-i{{\rm{\Omega }}}_{d}{\rho }_{31}+i{{\rm{\Omega }}}_{c}{\rho }_{24}-{\tilde{\gamma }}_{34}{\rho }_{34},$$

Here *ρ*_11_ + *ρ*_22_ + *ρ*_33_ + *ρ*_44_ = 1 and ${\rho }_{ij}={\rho }_{ji}^{\ast }$. Γ_*ij*_ is the natural decay rate between levels |*i*〉 and |*j*〉. We assume that the decay rates from levels |3〉 and |4〉 in the conduction band to levels |1〉 and |2〉 in the valence band are identical, there are, Γ_31_ = Γ_32_ and Γ_41_ = Γ_42_. There is also no decay in the valence band or conduction band, so we conclude that Γ_21_ = Γ_43_ = 0. ${{\rm{\Gamma }}}_{i}={\sum }_{j}^{i-1}{{\rm{\Gamma }}}_{ij}$ denotes the total decay rate of level |*i*〉. We define ${\tilde{\gamma }}_{12}$ = ${\gamma }_{12}+i({{\rm{\Delta }}}_{p}-{{\rm{\Delta }}}_{c})$, ${\tilde{\gamma }}_{13}$ = ${\gamma }_{13}+i{{\rm{\Delta }}}_{p}$, ${\tilde{\gamma }}_{14}$ = ${\gamma }_{14}+i{{\rm{\Delta }}}_{d}$, ${\tilde{\gamma }}_{23}$ = ${\gamma }_{23}+i{{\rm{\Delta }}}_{c}$, ${\tilde{\gamma }}_{24}$ = ${\gamma }_{24}+i({{\rm{\Delta }}}_{c}+{{\rm{\Delta }}}_{d}-{{\rm{\Delta }}}_{p})$, and ${\tilde{\gamma }}_{34}$ = ${\gamma }_{34}+i({{\rm{\Delta }}}_{d}-{{\rm{\Delta }}}_{p})$, where *γ*_*ij*_ = (Γ_*i*_ + Γ_*j*_)/2.

In such QW systems, ${{\rm{\Gamma }}}_{3}={{\rm{\Gamma }}}_{3l}+{{\rm{\Gamma }}}_{3}^{{\rm{dph}}}$ and ${{\rm{\Gamma }}}_{4}={{\rm{\Gamma }}}_{4l}+{{\rm{\Gamma }}}_{4}^{{\rm{dph}}}$, where Γ_3*l*_ and Γ_4*l*_ are the population decay rates of subbands |3〉 and |4〉 respectively, resulting from longitudinal-optical phonon emission events at low temperature, and ${{\rm{\Gamma }}}_{3}^{{\rm{dph}}}$ and ${{\rm{\Gamma }}}_{4}^{{\rm{dph}}}$ are the dephasing decay rates of quantum coherence due to electron–electron scattering, phonon scattering processes; and elastic interface roughness. Based on the previous studies, we assume that Γ_3*l*_ can be equal to Γ_4*l*_, and ${{\rm{\Gamma }}}_{3}^{{\rm{dph}}}$ can be equal to ${{\rm{\Gamma }}}_{4}^{{\rm{dph}}}$. Therefore, Γ_31_ = Γ_32_ = Γ_41_ = Γ_42_ = Γ can be presented. In addition, we assume there is no interference or dephasing between levels |3〉 and |4〉, which can be realized based on the appropriate reduction of the temperature^44^.

The susceptibility of the QW medium can be obtained through the expression $\chi$ = $N{\mu }_{13}/{\varepsilon }_{0}{E}_{p}\cdot {\rho }_{31}$ = $N{\mu }_{13}^{2}/2{\varepsilon }_{0}{{\rm{h}}{\rm{\Omega }}}_{p}\cdot {\rho }_{31}$, where *N* is the electron density of the QWs, and *ρ*_31_ can be obtained by Eq. (3). *χ* = *χ*_*R*_ + *iχ*_*I*_, where *χ*_*R*_ describes the dispersion properties of the probe field, while *χ*_*I*_ describes the absorption properties of the probe field with *χ*_*I*_ > 0 (*χ*_*I*_ < 0) indicating loss (gain).

The refractive index of the QW medium can be written as $n=\sqrt{{\varepsilon }_{c}+\chi }$. Here ${\varepsilon }_{c}={n}_{c}^{2}$ corresponding to the complex dielectric constant of the host QW medium, and *n*_*c*_ is its refractive index when light is far detuned from the resonance. *χ* denotes the change in the susceptibility^22,23^, which results from the coherent contract via the coupling and pump fields near the resonance. Generally, *χ* = *ε*_*c*_, then we have $n$ ≈ $\sqrt{{\varepsilon }_{c}}+\chi /22\sqrt{{\varepsilon }_{c}}$ = ${n}_{c}+\chi /2{n}_{c}$ = ${n}_{c}+{\chi }_{R}/2{n}_{c}+i{\chi }_{I}/2{n}_{c}$. We define the real and imaginary parts of the refractive index as *n*_*R*_ and *n*_*I*_, with *n*_*c*_ being the background index of the system. Thus, *n* = *n*_*c*_ + *n*_*R*_ + *in*_*I*_, where *n*_*R*_ = *χ*_*R*_/2*n*_0_ and *n*_*I*_ = *χ*_*I*_/2*n*_0_. To achieve *PT* symmetry, the condition of *n*_*R*_(*r*) = *n*_*R*_(−*r*) and *n*_*I*_(*r*) = −*n*_*I*_(−*r*) must be satisfied. And for simplicity, we use the unit $N{\mu }_{13}^{2}/4{\varepsilon }_{0}{\rm{h}}{n}_{0}$ in the following calculations.

## Results

### *PT*-symmetric optical waveguides

**Figure 2:**
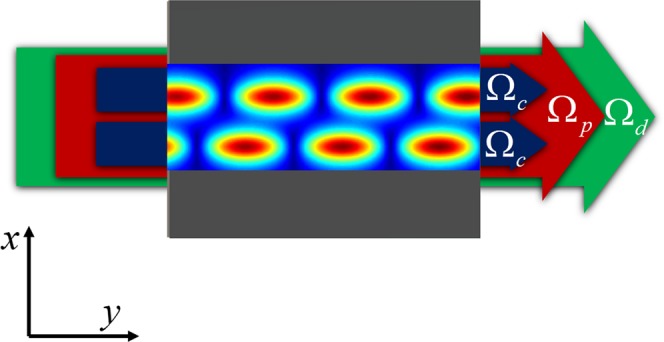
Schematic diagram of QW system for realizing *PT*-symmetric optical waveguides. *x* and *y* represent the transverse and longitudinal directions of laser beams.

In order to realize *PT*-symmetric optical waveguides, we use a pair of coupling laser beams to form two coupled waveguides. Such pair of beams propagate in QWs along *y* direction. Then, a pump and a probe laser beams with wider laser dimension propagate in the same direction as those of the coupling beams. The schematic diagram is shown in Fig. 2. The pair of coupling laser beams have an identical Gaussian intensity profile so that the total spatial intensity distribution of them varying in *x* direction is

4 $${I}_{c}(x)=A{e}^{\frac{-{(x-a)}^{2}}{2{\sigma }^{2}}}+A{e}^{\frac{-{(x+a)}^{2}}{2{\sigma }^{2}}},$$

where *A* and *a* are the constant and the half separation between the two waveguides respectively. $2\sqrt{2\,\mathrm{ln}\,2}\sigma$ can describe the full width at half maximum (FWHM) of the waveguides. By choosing two different detuning of the coupling fields, gain can be introduced to one waveguide and loss can be introduced to the other, even though other parameters are identical. In such consideration, the refractive index in each waveguide spatially varies only with the intensity of the coupling.

**Figure 3:**
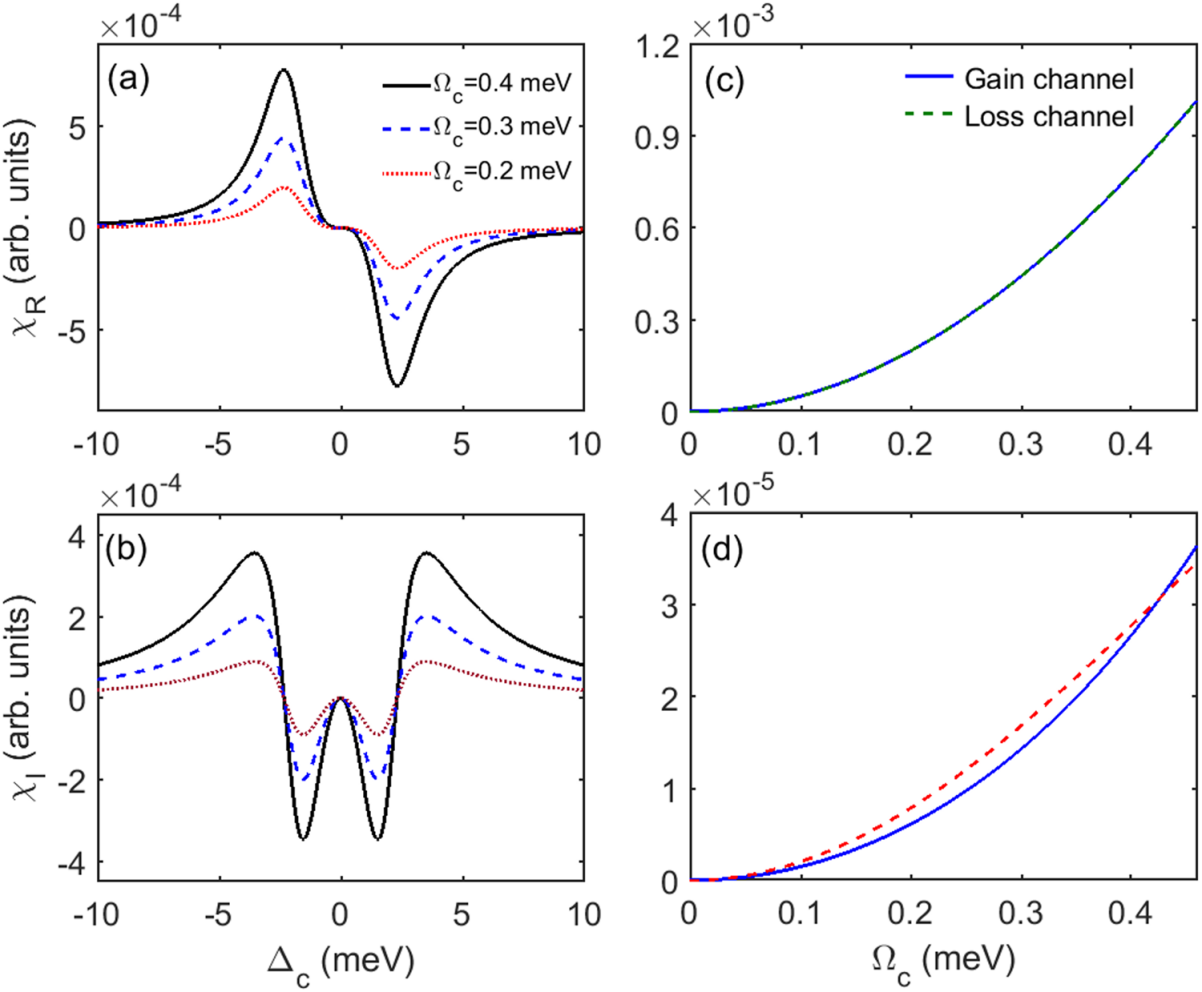
(**a**) Real part *χ*_*R*_ and (**b**) imaginary part *χ*_*I*_ of the susceptibility as a function of coupling detuning Δ_*c*_ for different coupling Rabi frequency, Ω_*c*_ = 0.4 meV (black solid line), Ω_*c*_ = 0.3 meV (blue dashed line), and Ω_*c*_ = 0.2 meV (red dotted line). (**c**) Real part *χ*_*R*_ and (**d**) imaginary part *χ*_*I*_ of the susceptibility as a function of coupling Rabi frequency Ω_*c*_ for different coupling detuning, Δ_*c*_ = −2.278 meV (blue solid line) and Δ_*c*_ = −2.360 meV (red dashed line). The other parameters are ${{\rm{\Gamma }}}_{3}^{{\rm{dph}}}={{\rm{\Gamma }}}_{4}^{{\rm{dph}}}=2.58{\rm{meV}}$, Γ_3*l*_ = Γ_4*l*_ = 2.07 meV, Γ_31_ = Γ_32_ = Γ_41_ = Γ_42_ = Γ = 2.325 meV, Ω_*p*_ = 0.01Γ, Ω_*d*_ = 2Γ, Δ_*p*_ = Δ_*d*_ = 0.

To realize gain and absorption in two waveguides simultaneously, different detuning of the coupling fields need to be found. For sake of it, we calculate the real (dispersion) *χ*_*R*_ and imaginary (gain or absorption) *χ*_*I*_ parts of the susceptibility as a function of Δ_*c*_, and show the results in Fig. 3(a,b), respectively. The intensity of the coupling field in each waveguide is corresponding to Gaussian profile, so *χ*_*I*_ needs to get larger with increasing coupling intensity. For this reason, we only consider a negative value of the coupling detuning. It can be seen from the figures that for different value of Ω_*c*_, *χ*_*R*_ reaches to the maximum value, and *χ*_*I*_ is close to zero at Δ_*c*_ = −2.325Γ. At the vicinity of the zero point, gain is obtained on the left side, and absorption is abstained on the right side. This property called refractive index enhancement with vanishing absorption, has been demonstrated in the early papers^45–49^.

In order to realize gain and loss in two waveguides simultaneously by using two different coupling detuning and maintain other parameters identical, the relation between the susceptibility and the coupling intensity needs to be established. Therefore, we choose Δ_*c*_ = −2.360Γ and Δ_*c*_ = −2.278Γ in two waveguides, and draw *χ*_*R*_ and *χ*_*I*_ as a function of Ω_*c*_ shown in Fig. 3(c,d). It can be seen that with selected coupling detuning, the curves of *χ*_*R*_ are identical, and the curves of *χ*_*I*_ are matched with slight difference, respectively. It should be noted that, in Fig. 3(d) we flipped the curve of the gain one by multiplying a minus sign to compare the two curves of gain and loss directly.

**Figure 4:**
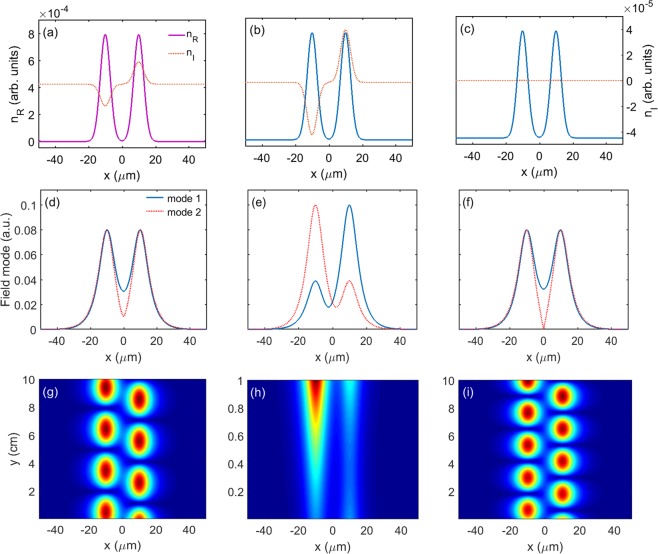
Real part *n*_*R*_ and imaginary part *n*_*I*_ of the refractive index as a function of position *x* for different cases, (**a**) Δ_*c*_ = −2.350 meV (loss waveguide) and Δ_*c*_ = −2.300 meV (gain waveguide), representing the condition of below threshold, (**b**) Δ_*c*_ = −2.386 meV (loss waveguide) and Δ_*c*_ = −2.266 meV (gain waveguide), representing the condition of above threshold, (**c**) Δ_*c*_ = −2.325 meV (both waveguides), representing the condition of non-*PT* symmetry, respectively. (**d**–**f**) are filed mode of the probe laser beam according to (**a**–**c**). (**g**–**i**) are propagation properties of the probe laser beam according to (**a**–**c**). The other parameters are the same as in Fig. 3.

Based on the above analysis, firstly, we demonstrate the possibility of realizing spatial modulation of the refractive index. Making use of Eq. (4), and choosing the FWHM ($2\sqrt{2\,\mathrm{ln}\,2}\sigma$) as 7 *μ*m and the separation between the two waveguides as 20 *μ*m, we plot the real *n*_*R*_ and the imaginary *n*_*I*_ parts of the refractive index as a function of *x* shown in Fig. 4(a–c). It can be seen from the figures that the real part of the refractive index *n*_*R*_ is an even function of *x*, while that of imaginary part *n*_*I*_ is an odd function of *x*. By changing the electron intensity of QW, The absolute values of *n*_*R*_ and *n*_*I*_ can be modified with equal scale simultaneously with the changes of the electron intensity of QW.

By controlling the ratio of the real and imaginary parts of the refractive index, we can adjust the condition of the system below or above the *PT*-symmetric threshold. It can be seen from Fig. 3(a,b) that *χ*_*R*_ changes less than *χ*_*I*_ at the vicinity of zero point (Δ_*c*_ = −2.325Γ). Therefore, we can alter the coupling detuning in the waveguides to control the ratio. For instance, with different values of the coupling detuning, the ratio of the real and imaginary parts is 12 and 80, and the system can be operated below (Fig. 4(a)) or above (Fig. 4(b)) the *PT*-symmetric threshold respectively. In addition, when the coupling detuning used in both waveguides is −2.325Γ, the system even becomes a non-symmetric one with much larger ratio (Fig. 4(c)).

Secondly, we present the field modes in the waveguides in Fig. 4(d–f) corresponding to Fig. 4(a–c), respectively. When the condition of the system is below the threshold (Fig. 4(a)), the *PT* symmetry condition is satisfied, the eigenvalues should be real, and the field modes should be symmetric. However, because of the imperfect *PT* symmetry condition, the eigenvalues have a very small imaginary part, and the two field modes are slightly asymmetric, as shown in Fig. 4(d). For the case of above the threshold (Fig. 4(b)), that is, the *PT* symmetry is broken, the eigenvalues become complex, where the imaginary part of the refractive index represents the gain or loss for each filed mode. Figure 4(e) illustrates that, the light modes become strongly asymmetric. For the non-symmetric one (Fig. 4(c)), the light modes are perfectly symmetric, which is shown in Fig. 4(f).

Thirdly, we show in Fig. 4(g–i) the propagation characteristics of the probe beam according to the condition of Fig. 4(a–c) respectively. When the condition of the system is below threshold, the probe beam oscillates periodically between the two waveguides, as show in Fig. 4(g). When the case is above the threshold, it can be seen from Fig. 4(h) that an exponentially growing mode occurs, which signifies the onset of *PT* symmetry breaking. For the passive waveguides, the probe beam also oscillates periodically (Fig. 4(i)), which is similar to the case of below threshold. However, the oscillation period in Fig. 4(i) is shorter than that of in Fig. 4(g).

In a practical system, it is very difficult to realize perfect *PT*-symmetric condition. So, we evaluate the degrees of asymmetry of the system by analyzing the asymmetry function of Δ(*x*) = *n*(*x*) − n*(−*x*), and find that the degree of asymmetry is very small. Therefore, it is manageable and has little impact on practical experiments.

### *PT*-symmetric optical lattices—Type I

**Figure 5:**
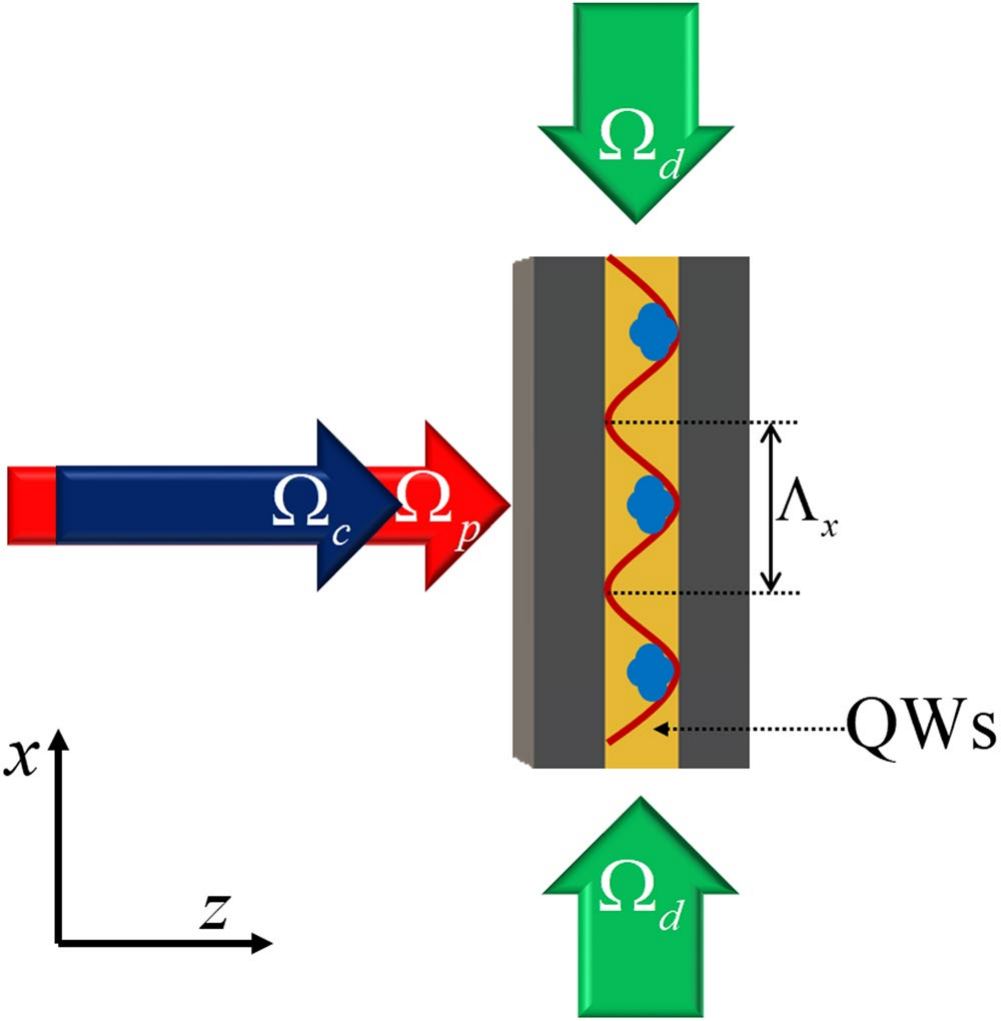
Schematic diagram of QW system for realizing *PT*-symmetric optical lattices. Electron density of QW is modulated in *x* direction.

In this part, we first consider an 1D optical lattices of period Λ_*x*_ along *x* direction, and in each lattices the electron density of QWs is spatially modulated. The electron density in the *i*th trap can exhibit a Gaussian distribution ${N}_{i}(x,y)=N{e}^{-{(x-{x}_{i})}^{2}/{\sigma }_{x}^{2}}$, where *N* is the electron density of a homogeneous QW sample, *x*_*i*_ is the *i*th trap center, and *σ*_*x*_ is the half width. The modulation of electron density of QWs can be achieved by using high order surface grating^50,51^. We further consider a pump laser field with periodically modulated intensity ${{\rm{\Omega }}}_{d}={{\rm{\Omega }}}_{d0}+\delta {{\rm{\Omega }}}_{d}\,\sin \,[2\pi (x-{x}_{i})/{{\rm{\Lambda }}}_{x}]$, which can be easily achieved in experiment by using an imperfect standing-wave (SW) field with unequal forward and backward components. We show the schematic diagram of QW system in Fig. 5. The probe field and coupling filed propagate along *z* direction, and the SW pump fields propagate along *x* direction.

**Figure 6:**
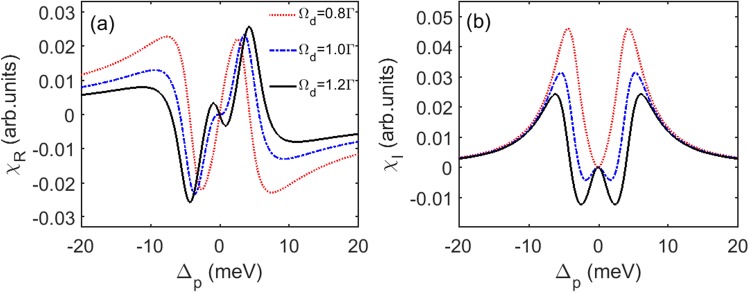
(**a**) Real part *χ*_*R*_ and (**b**) imaginary part *χ*_*I*_ of the susceptibility as a function of probe detuning Δ_*p*_ for different pump Rabi frequency, Ω_*d*_ = 0.8Γ (red dotted line), Ω_*d*_ = Γ (blue dashed line), and Ω_*d*_ = 1.2Γ (black solid line). The parameters are Ω_*c*_ = Γ and Δ_*c*_ = Δ_*d*_ = 0. The other parameters are the same as in Fig. 3.

First, we calculate the real *χ*_*R*_ and imaginary *χ*_*I*_ parts of the susceptibility as a function of Δ_*p*_ for varying value of Ω_*d*_ by solving Eq. (3) without modulation of pump intensity or the electron density, and the corresponding results are shown in Fig. 6. It can be seen from red dotted line in Fig. 6(b) that when Ω_*d*_ = 0.8Γ, a typical EIT with positive value (loss) is realized. With increasing value of Ω_*d*_, at both sides of the EIT windows, *χ*_*I*_ becomes positive (gain), which is knew as coherent Raman gain without population inversion [blue dashed line and black solid line in Fig. 6(b)]. Meanwhile, *χ*_*R*_ changes form positive dispersion to negative dispersion in the vicinity of EIT window, as shown in Fig. 6(a). The figures indicate that it is possible to realize *PT* symmetry by choosing suitable modulations.

**Figure 7:**
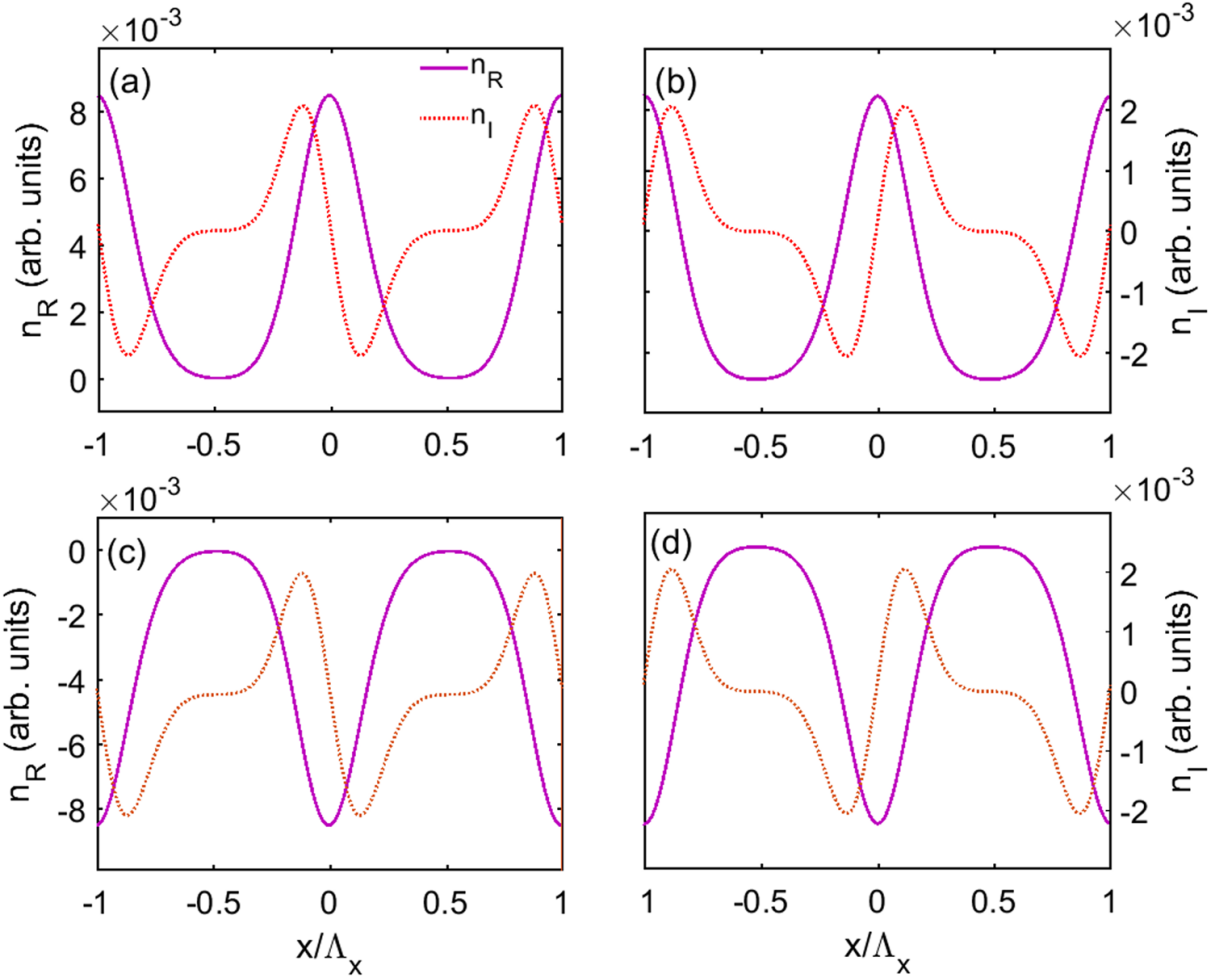
Real part *n*_*R*_ and imaginary part *n*_*I*_ of the refractive index as a function of position *x* for unchanged absolute value and different signs of Δ_*p*_ and *δ*Ω_*d*_, respectively, (**a**) Δ_*p*_ = 2.592 meV and *δ*Ω_*d*_ = 0.1Γ, (**b**) Δ_*p*_ = 2.592 meV and *δ*Ω_*d*_ = −0.1Γ, (**c**) Δ_*p*_ = −2.592 meV and *δ*Ω_*d*_ = 0.1Γ, (**d**) Δ_*p*_ = −2.592 meV and *δ*Ω_*d*_ = −0.1Γ. The parameters are Ω_*d*0_ = Γ. The other parameters are the same as in Fig. 6.

Then, with modulation of the pump intensity and electron density, we calculate the real (*n*_*R*_) and imaginary parts (*n*_*I*_) of the refractive index as a function of *x* and show the results in Fig. 7. Figure 7(a) shows the condition of Δ_*p*_ = 2.592 meV and *δ*Ω_*d*_ = 0.1Γ. The value of Δ_*p*_ is chosen according to blue dashed line in Fig. 6(b), where *χ*_*I*_ has zero value around Δ_*p*_ = 2.592 meV, and thus it exhibits gain or absorption if Δ_*p*_ is slightly tuned away from this point. It can be seen that *n*_*R*_ is an even function of the lattice position *x*, while *n*_*I*_ is an odd function of lattice position *x* with balance gain and loss. Such results clearly indicate that *PT* symmetry is built in QW system by modulating the pump field and the electron density.

In Figs 7(b) and 6(d), we further show the cases for the same absolute value of Δ_*p*_ and *δ*Ω_*d*_ as that of in Fig. 7(a), but with different sign of them. The results show that the *PT*-symmetric properties are maintained in all cases. However, the relation between *n*_*R*_ and *n*_*I*_ are different. It is found that positive (negative) value of Δ_*p*_ results in the positive (negative) value of *n*_*R*_ and have no effect on the profile of *n*_*I*_. On the other hand, the sign of *δ*Ω_*d*_ determines the position of gain and loss in each period, for instance, with positive (negative) value of *δ*Ω_*d*_, *n*_*I*_ is gain (loss) in one half period −0.5 ≤ *x*/Λ_*x*_ ≤ 0. The understanding of impacts of the parameters on spatial features of the refractive index is important because of its potential application, such as asymmetric light diffraction^52,53^.

**Figure 8:**
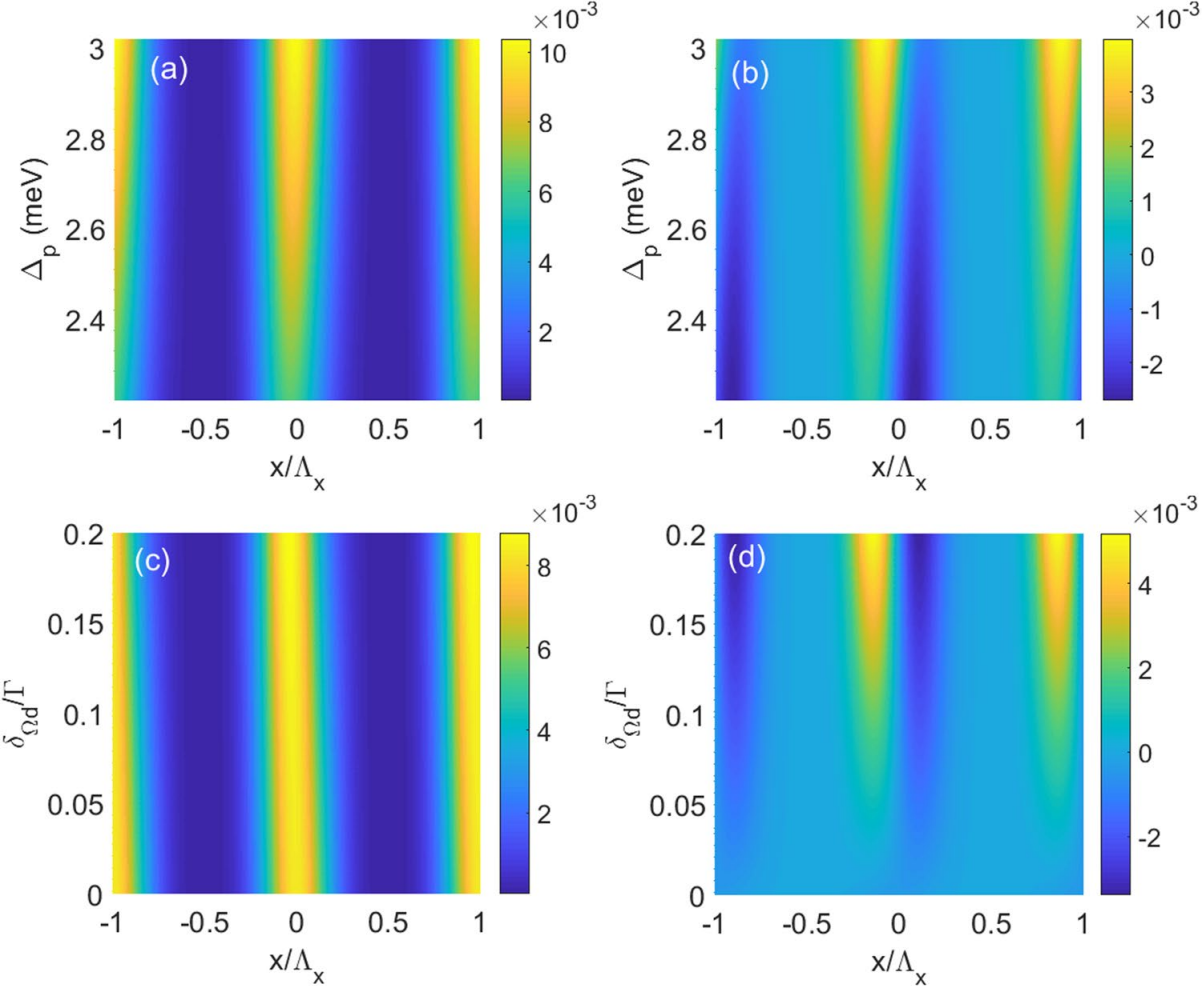
(**a**) Real part *n*_*R*_ and (**b**) imaginary part *n*_*I*_ of the refractive index as functions of position *x* and probe detuning Δ_*p*_. The parameters are *δ*Ω_*d*_ = 0.1Γ. (**c**) Real part *n*_*R*_ and (**d**) imaginary part *n*_*I*_ of the refractive index as functions of position *x* and modulation intensity *δ*Ω_*d*_. The parameters are Δ_*p*_ = 2.592 meV. The other parameters are the same as in Fig. 7.

We next check what will happen if the absolute value of Δ_*p*_ and *δ*Ω_*d*_ is changed. To do so, we plot 2D *n*_*R*_ and *n*_*I*_ as functions of *x* and Δ_*p*_ in Fig. 8(a,b), and as functions of *x* and *δ*Ω_*d*_ in Fig. 8(c,d), respectively. The figures clearly show that *n*_*R*_ and *n*_*I*_ are modulated along *x* direction for varying Δ_*p*_ or *δ*Ω_*d*_. In addition, the profile of *n*_*I*_ is much more sensitive to the parameters than that of *n*_*R*_. When Δ_*p*_ or *δ*Ω_*d*_ is detuned from the value used in Fig. 7(a), through *n*_*R*_ is maintained an even property, *n*_*I*_ losses odd property, where the loss in one half period is not equal to the gain in the other half. Therefore, *PT* symmetry is destroyed in QW system because of the nonlinear response of *n*_*I*_ to Δ_*p*_ or *δ*Ω_*d*_.

Last, our goal is to realize 2D modulation of refractive index as functions of *x* and *y*, and this can be achieved by 2D modulation of the electron density and pump field, which are

5a $${N}_{i}(x,y)=N{e}^{-{(x-{x}_{i})}^{2}/{\sigma }_{x}^{2}-{(y-{y}_{i})}^{2}/{\sigma }_{y}^{2}},$$

5b $${{\rm{\Omega }}}_{d}={{\rm{\Omega }}}_{d0}+0.5\,\delta {{\rm{\Omega }}}_{d}\{\sin \,[2\pi (x-{x}_{i})/{{\rm{\Lambda }}}_{x}]+\,\sin \,[2\pi (y-{y}_{i})/{\Lambda }_{y}]\},$$

**Figure 9:**
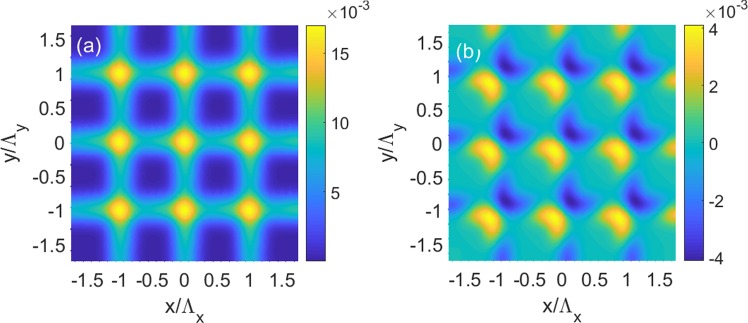
(**a**) Real part *n*_*R*_ and (**b**) imaginary part *n*_*I*_ of the refractive index as functions of position *x* and position y. The parameters are Δ_*p*_ = 2.592 meV and *δ*Ω_*d*_ = 0.1Γ. The other parameters are the same as in Fig. 7.

We plot in Fig. 9(a,b) the top view of *n*_*R*_ and *n*_*I*_ as functions of *x* and *y* using the same parameters in Fig. 7(a), respectively. The results clearly show that *n*_*R*_ is an even functions of *x* and *y*, while *n*_*I*_ is an odd functions of *x* and *y*, therefore, 2D *PT*-symmetric optical lattices is realized in QW systems.

### *PT*-symmetric optical lattices—Type II

**Figure 10:**
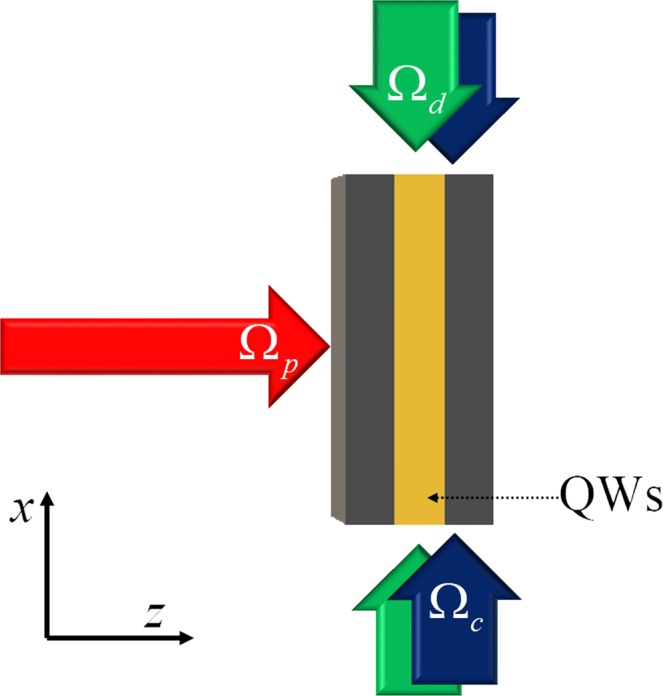
Schematic diagram of QW system for realizing *PT*-symmetric optical lattices.

In this part, we show that *PT* symmetry can also be achieved by modulating both pump and coupling fields. The schematic diagram of the system is shown in Fig. 10, where the probe field propagates along *z* direction, and the SW coupling and SW pump fields propagate along *x* direction. Therefore, we have modulated coupling and pump fields, ${{\rm{\Omega }}}_{c}$ = ${{\rm{\Omega }}}_{c0}+\delta {{\rm{\Omega }}}_{c}\,\cos \,[2\pi (x-{x}_{i}){/{\rm{\Lambda }}}_{x}]$, ${{\rm{\Omega }}}_{d}$ = ${{\rm{\Omega }}}_{d0}+\delta {{\rm{\Omega }}}_{d}\,\sin \,[2\pi (x-{x}_{i}){/{\rm{\Lambda }}}_{x}]$.

**Figure 11:**
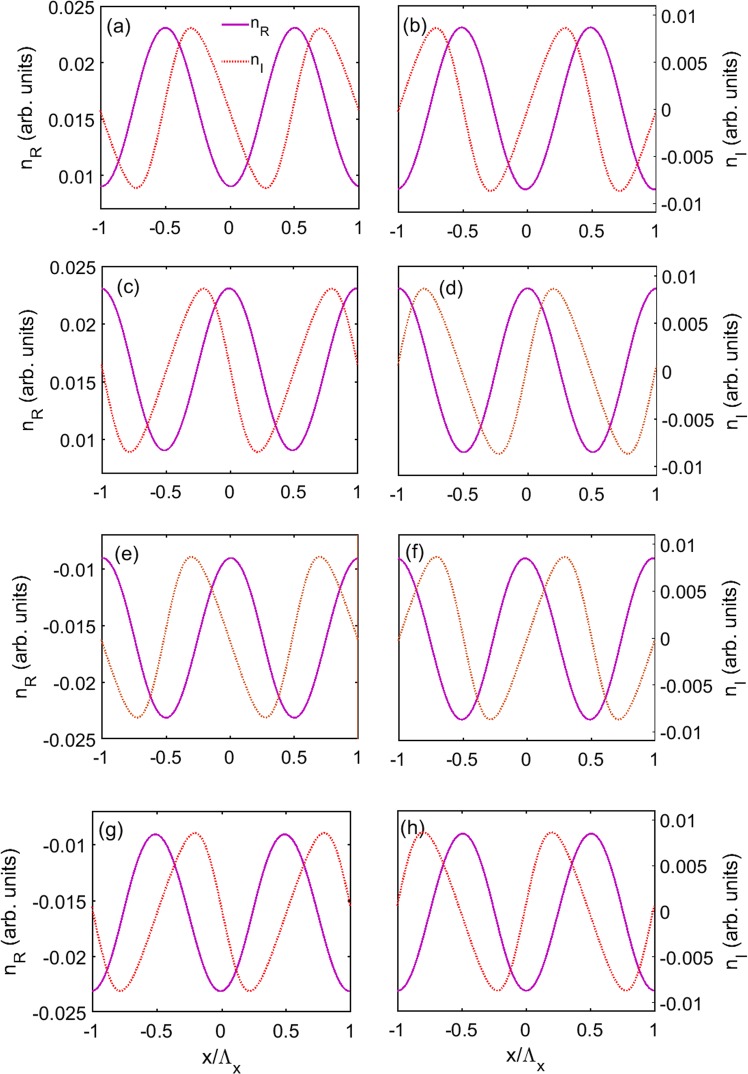
Real part *n*_*R*_ and imaginary part *n*_*I*_ of the refractive index as a function of position *x* for unchanged absolute value and different signs of Δ_*p*_, *δ*Ω_*c*_ and *δ*Ω_*d*_, respectively, (**a**) Δ_*p*_ = 2.525 meV, *δ*Ω_*c*_ = 0.3Γ and *δ*Ω_*d*_ = 0.1Γ, (**b**) Δ_*p*_ = 2.525 meV, *δ*Ω_*c*_ = 0.3Γ and *δ*Ω_*d*_ = −0.1Γ, (**c**) Δ_*p*_ = 2.525 meV, *δ*Ω_*c*_ = −0.3Γ and *δ*Ω_*d*_ = 0.1Γ, (**d**) Δ_*p*_ = 2.525 meV, *δ*Ω_*c*_ = −0.3Γ and *δ*Ω_*d*_ = −0.1Γ, (**e**) Δ_*p*_ = −2.525 meV, *δ*Ω_*c*_ = 0.3Γ and *δ*Ω_*d*_ = 0.1Γ, (**f**) Δ_*p*_ = −2.525 meV, *δ*Ω_*c*_ = 0.3Γ and *δ*Ω_*d*_ = −0.1Γ, (**g**) Δ_*p*_ = −2.525 meV, *δ*Ω_*c*_ = −0.3Γ and *δ*Ω_*d*_ = 0.1Γ, (**h**) Δ_*p*_ = −2.525 meV, *δ*Ω_*c*_ = −0.3Γ and *δ*Ω_*d*_ = −0.1Γ. The parameters are Ω_*c*0_ = 3Γ and Ω_*d*0_ = Γ. The other parameters are the same as in Fig. 6.

First, we plot the real and imaginary parts of the refractive index (*n*_*R*_ and *n*_*I*_) as a function of *x* in Fig. 11 for different cases. Figure 11(a) shows the condition of Δ_*p*_ = 2.592 meV and the modulation intensities *δ*Ω_*c*_ = 0.3Γ and *δ*Ω_*d*_ = 0.1Γ. It can be seen from figure that the *PT* symmetry is appears, where *n*_*R*_ is an even function of *x*, and *n*_*I*_ is an odd function of *x*. The relations of *n*_*R*_ and *n*_*I*_ can also be modified by changing the sign of the detuning Δ_*p*_ and the modulation intensities *δ*Ω_*c*_ and *δ*Ω_*d*_, and the changing of their sign will not destroy the *PT* symmetry. There are eight kinds of combinations of these three parameters, corresponding to eight kinds of spatial refractive index, and we plot the other seven kinds in Fig. 11(b–h). It can be found that Δ_*p*_, *δ*Ω_*c*_ or *δ*Ω_*d*_ have different impacts on the features of *n*_*R*_ and *n*_*I*_. More specifically, Δ_*p*_ determines *n*_*R*_ being positive or negative, and *δ*Ω_*c*_ determines the monotonicity of *n*_*R*_, and *δ*Ω_*d*_ determines the positions of the gain and loss in one period.

**Figure 12:**
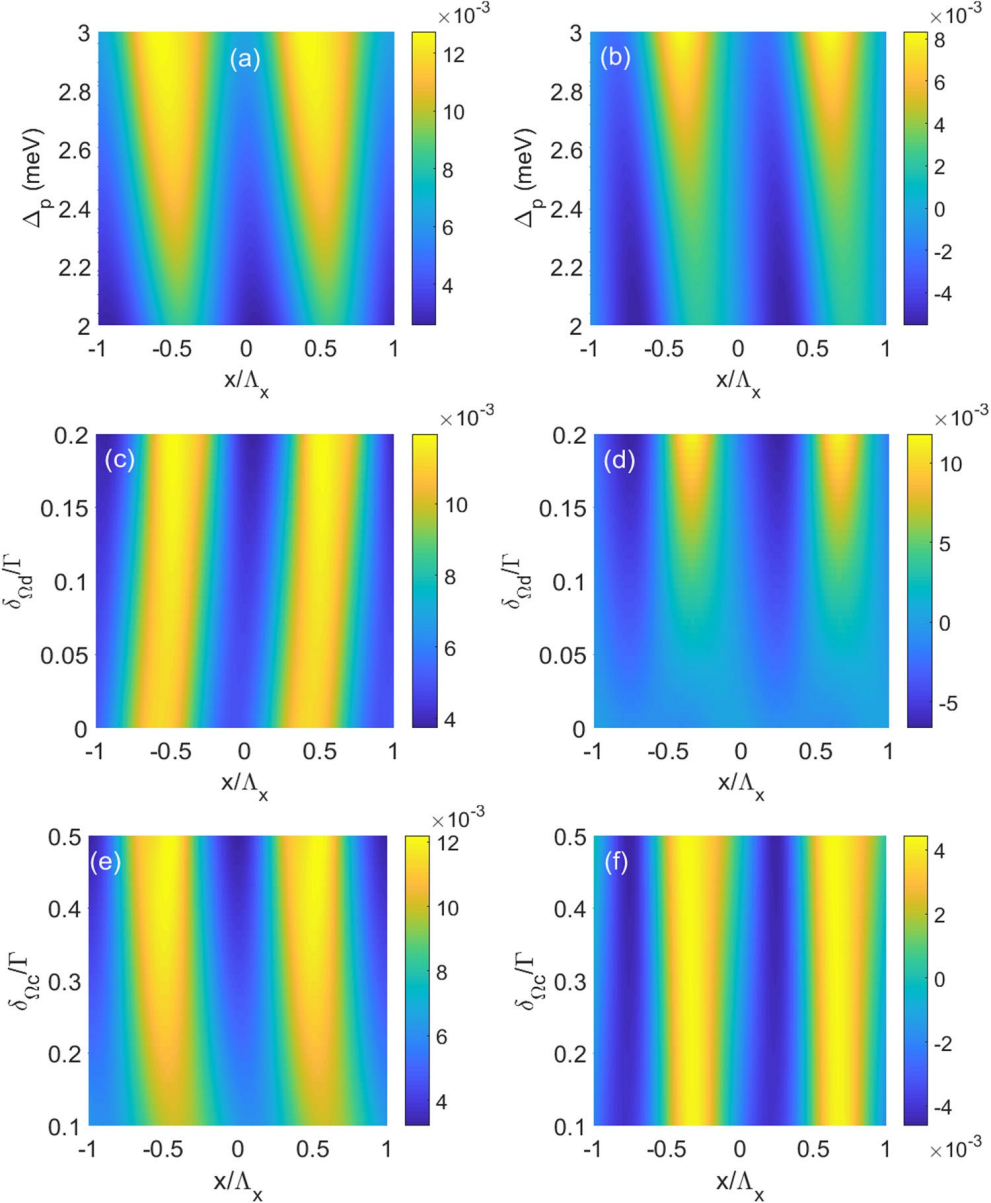
(**a**) Real part *n*_*R*_ and (**b**) imaginary part *n*_*I*_ of the refractive index as functions of position *x* and probe detuning Δ_*p*_. The parameters are *δ*Ω_*c*_ = 0.3Γ and *δ*Ω_*d*_ = 0.1Γ. (**c**) Real part *n*_*R*_ and (**d**) imaginary part *n*_*I*_ of the refractive index as functions of position *x* and modulation intensity *δ*Ω_*d*_. The parameters are Δ_*p*_ = 2.525 meV and *δ*Ω_*c*_ = 0.3Γ. (**e**) Real part *n*_*R*_ and (**f**) imaginary part *n*_*I*_ of the refractive index as functions of position *x* and modulation intensity *δ*Ω_*c*_. The parameters are Δ_*p*_ = 2.525 meV and *δ*Ω_*d*_ = 0.1Γ. The other parameters are the same as in Fig. 11.

We also check the impact of absolute value of Δ_*p*_, *δ*Ω_*c*_ or *δ*Ω_*d*_ on the properties of *PT*-symmetric system. Therefore, we plot 2D *n*_*R*_ and *n*_*I*_ as functions of *x* and Δ_*p*_ in Fig. 12(a,b), as functions of *x* and *δ*Ω_*d*_ in Fig. 12(c,d), and as functions of *x* and *δ*Ω_*c*_ in Fig. 12(e,f), respectively. The figures clearly show that *n*_*R*_ and *n*_*I*_ are modulated along *x* direction for varying Δ_*p*_, *δ*Ω_*c*_ or *δ*Ω_*d*_. However, when these parameters are detuned form the value used in Fig. 11(a), the system will loss the *PT*-symmetric properties.

Last, we show that it is possible to realize 2D *PT*-symmetric optical lattices by applying 2D modulation of the pump and coupling laser fields, which are

6a $${{\rm{\Omega }}}_{c}={{\rm{\Omega }}}_{c0}+0.5\,\delta {{\rm{\Omega }}}_{c}\{\cos \,[2\pi (x-{x}_{i})/{{\rm{\Lambda }}}_{x}]+\,\cos \,[2\pi (y-{y}_{i})/{{\rm{\Lambda }}}_{y}]\},$$

6b $${{\rm{\Omega }}}_{d}={{\rm{\Omega }}}_{d0}+0.5\,\delta {{\rm{\Omega }}}_{d}\{\sin \,[2\pi (x-{x}_{i})/{{\rm{\Lambda }}}_{x}]+\,\sin \,[2\pi (y-{y}_{i}){/{\rm{\Lambda }}}_{y}]\},$$

**Figure 13:**
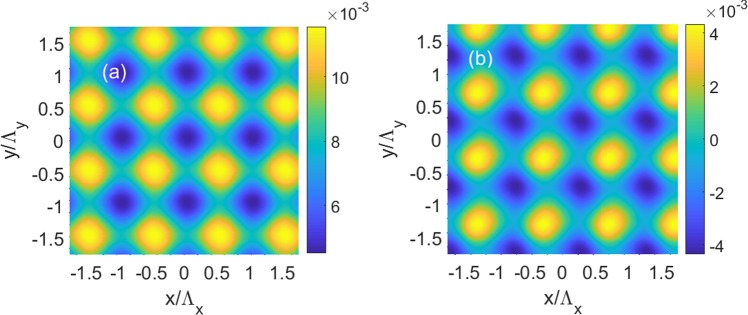
(**a**) Real part *n*_*R*_ and (**b**) imaginary part *n*_*I*_ of the refractive index as functions of position *x* and position y. The parameters are Δ_*p*_ = 2.525 meV, *δ*Ω_*c*_ = 0.3Γ and *δ*Ω_*d*_ = 0.1Γ. The other parameters are the same as in Fig. 11.

Using the same parameters in Fig. 11(a), we calculate *n*_*R*_ and *n*_*I*_ as functions of *x* and *y*, and show the tope view of *n*_*R*_ and *n*_*I*_ as functions of *x* and *y*in Fig. 13(a,b), respectively. The results indicate that 2D *PT*-symmetric optical lattices can be achieved, with *n*_*R*_ being an even functions of *x* and *y*, and *n*_*I*_ being an odd functions of *x* and *y*, respectively.

## Conclusion

In conclusion, we have demonstrated that coherent asymmetric double semiconductor QWs can be an ideal candidate for studying *PT* symmetry. We have showed that *PT*-symmetric optical waveguides can be realized by using two coupling fields with different detuning, 1D and 2D *PT*-symmetric optical lattices can be realized by spatial modulation of pump laser fields and the electron density of QWs, or by spatial modulation of pump and coupling fields. In addition, the *PT*-symmetric properties realized in QW systems, such as the relation between the real and the imaginary parts of the complex refractive indices, can be controlled by changing the laser fields and the parameters of QWs.

## References

[1] Bender CM, Boettcher S (1998). Real spectra in non-Hermitian Hamiltonians having PT symmetry. Physical Review Letters.

[2] El-Ganainy R, Makris KG, Christodoulides DN, Musslimani ZH (2007). Theory of coupled optical PT-symmetric structures. Opt. Lett..

[3] Ruter CE (2010). Observation of parity-time symmetry in optics. Nat Phys.

[4] Guo A (2009). Observation of PT-Symmetry Breaking in Complex Optical Potentials. Physical Review Letters.

[5] Regensburger A (2012). Parity-time synthetic photonic lattices. Nature.

[6] Wimmer M (2015). Observation of optical solitons in PT-symmetric lattices. Nature Communications.

[7] Peng B (2014). Parity-time-symmetric whispering-gallery microcavities. Nat Phys.

[8] Chang L (2014). Parity-time symmetry and variable optical isolation in active-passive-coupled microresonators. Nat Photon.

[9] Feng L (2013). Experimental demonstration of a unidirectional reflectionless parity-time metamaterial at optical frequencies. Nat Mater.

[10] Lin Z (2011). Unidirectional Invisibility Induced by PT-Symmetric Periodic Structures. Physical Review Letters.

[11] Longhi S (2010). PT-symmetric laser absorber. Physical Review A.

[12] Chong YD, Ge L, Cao H, Stone AD (2010). Coherent Perfect Absorbers: Time-Reversed Lasers. Physical Review Letters.

[13] Hodaei H, Miri M-A, Heinrich M, Christodoulides DN, Khajavikhan M (2014). Parity-time-symmetric microring lasers. Science.

[14] Feng L, Wong ZJ, Ma R-M, Wang Y, Zhang X (2014). Single-mode laser by parity-time symmetry breaking. Science.

[15] Jing H (2014). PT-Symmetric Phonon Laser. Physical Review Letters.

[16] Fleury R, Sounas D, Alù A (2015). An invisible acoustic sensor based on parity-time symmetry. Nature Communications.

[17] Musslimani ZH, Makris KG, El-Ganainy R, Christodoulides DN (2008). Optical Solitons in PT Periodic Potentials. Physical Review Letters.

[18] Longhi S (2009). Bloch Oscillations in Complex Crystals with PT-Symmetry. Physical Review Letters.

[19] Liang GQ, Chong YD (2013). Optical Resonator Analog of a Two-Dimensional Topological Insulator. Physical Review Letters.

[20] Fejer MM, Yoo SJB, Byer RL, Harwit A, HarrisJr JS (1989). Observation of extremely large quadratic susceptibility at 9.6–10.8 um in electric-field-biased AlGaAs quantum wells. Physical Review Letters.

[21] Sirtori C, Capasso F, Sivco DL, Cho AY (1992). Giant, triply resonant, third-order nonlinear susceptibility in coupled quantum wells. Physical Review Letters.

[22] Zhang L (2005). Electric field effect on the linear and nonlinear intersubband refractive index changes in asymmetrical semiparabolic and symmetrical parabolic quantum wells. Superlattices and Microstructures.

[23] Kuhn KJ, Iyengar GU, Yee S (1991). Free carrier induced changes in the absorption and refractive index for intersubband optical transitions in AlxGa1−xAs/GaAs/AlxGa1−xAs quantum wells. Journal of Applied Physics.

[24] Frogley MD, Dynes JF, Beck M, Faist J, Phillips CC (2006). Gain without inversion in semiconductor nanostructures. Nature Materials.

[25] West LC, Eglash SJ (1985). First observation of an extremely large‐dipole infrared transition within the conduction band of a GaAs quantum well. Applied Physics Letters.

[26] Silvestri L, Bassani F, Czajkowski G, Davoudi B (2002). Electromagnetically induced transparency in asymmetric double quantum wells. The European Physical Journal B - Condensed Matter and Complex Systems.

[27] Phillips M, Wang H (2003). Electromagnetically induced transparency due to intervalence band coherence in a GaAs quantum well. Opt. Lett..

[28] Yang W-X, Lee R-K (2008). Controllable entanglement and polarization phase gate in coupled double quantum-well structures. Opt. Express.

[29] Zhu C, Huang G (2009). Slow-light solitons in coupled asymmetric quantum wells via interband transitions. Physical Review B.

[30] Luo XQ, Wang DL, Zhang ZQ, Ding JW, Liu WM (2011). Nonlinear optical behavior of a four-level quantum well with coupled relaxation of optical and longitudinal phonons. Physical Review A.

[31] Sheng J, Miri M-A, Christodoulides DN, Xiao M (2013). PT-symmetric optical potentials in a coherent atomic medium. Physical Review A.

[32] Li H-j, Dou J-p, Huang G (2013). PT symmetry via electromagnetically induced transparency. Opt. Express.

[33] Hang C, Huang G, Konotop VV (2013). PT Symmetry with a System of Three-Level Atoms. Physical Review Letters.

[34] Wang X, Wu J-H (2016). Optical PT-symmetry and PT-antisymmetry in coherently driven atomic lattices. Opt. Express.

[35] Zhang Z (2016). Observation of Parity-Time Symmetry in Optically Induced Atomic Lattices. Physical Review Letters.

[36] Kang H, Zhu Y (2003). Observation of Large Kerr Nonlinearity at Low Light Intensities. Physical Review Letters.

[37] Schmidt H, Imamoglu A (1996). Giant Kerr nonlinearities obtained by electromagnetically induced transparency. Opt. Lett..

[38] Sheng J, Yang X, Wu H, Xiao M (2011). Modified self-Kerr-nonlinearity in a four-level N-type atomic system. Physical Review A.

[39] Abdullaev FK, Kartashov YV, Konotop VV, Zezyulin DA (2011). Solitons in PT-symmetric nonlinear lattices. Physical Review A.

[40] Hang C, Zezyulin DA, Konotop VV, Huang G (2013). Tunable nonlinear parity-time-symmetric defect modes with an atomic cell. Opt. Lett..

[41] Ramezani H, Kottos T, El-Ganainy R, Christodoulides DN (2010). Unidirectional nonlinear PT-symmetric optical structures. Physical Review A.

[42] Roskos HG (1992). Coherent submillimeter-wave emission from charge oscillations in a double-well potential. Physical Review Letters.

[43] Nikonov DE, Imamoğlu A, Butov LV, Schmidt H (1997). Collective Intersubband Excitations in Quantum Wells: Coulomb Interaction versus Subband Dispersion. Physical Review Letters.

[44] Wu J-H (2005). Ultrafast All Optical Switching via Tunable Fano Interference. Physical Review Letters.

[45] Scully MO (1991). Enhancement of the index of refraction via quantum coherence. Physical Review Letters.

[46] Fleischhauer M (1992). Resonantly enhanced refractive index without absorption via atomic coherence. Physical Review A.

[47] Zibrov AS (1996). Experimental Demonstration of Enhanced Index of Refraction via Quantum Coherence in Rb. Physical Review Letters.

[48] Yavuz DD (2005). Refractive Index Enhancement in a Far-Off Resonant Atomic System. Physical Review Letters.

[49] Proite NA, Unks BE, Green JT, Yavuz DD (2008). Refractive Index Enhancement with Vanishing Absorption in an Atomic Vapor. Physical Review Letters.

[50] Dridi K, Benhsaien A, Zhang J, Hinzer K, Hall TJ (2014). Narrow linewidth two-electrode 1560 nm laterally coupled distributed feedback lasers with third-order surface etched gratings. Opt. Express.

[51] Gao F (2018). Study of gain-coupled distributed feedback laser based on high order surface gain-coupled gratings. Optics Communications.

[52] Liu Y-M, Gao F, Fan C-H, Wu J-H (2017). Asymmetric light diffraction of an atomic grating with PT symmetry. Opt. Lett..

[53] Shui T, Yang W-X, Liu S, Li L, Zhu Z (2018). Asymmetric diffraction by atomic gratings with optical PT symmetry in the Raman-Nath regime. Physical Review A.

